# Long‐Term Safety Evaluations in the Presence of Switching: Evaluation of Two Approaches

**DOI:** 10.1002/pst.70039

**Published:** 2025-09-26

**Authors:** Sandra Schmeller, Rima Izem, Pedro Lopez Romero, Valentine Jehl

**Affiliations:** ^1^ Institute of Statistics Ulm University Ulm Germany; ^2^ Novartis Pharma AG Basel Switzerland; ^3^ Novartis Pharma AG Madrid Spain

**Keywords:** Illness‐death model, long‐term safety, semi‐ and non‐parametric survival analysis, timescale, treatment switching

## Abstract

Evaluating the long‐term safety of approved drugs for chronic indications is essential. This assessment ensures that the benefits continue to outweigh the risks beyond the follow‐up observed in the clinical trials supporting market authorization. Consequently, these evaluations are mandated or recommended by multiple regulatory agencies and necessitate collecting and analyzing data from longitudinal cohorts in real‐world settings post‐market authorization. One challenge in analyzing and interpreting results using these sources is the complexity of long‐term real‐world drug utilization patterns in a competitive landscape, including switching between multiple drugs. Several methods have been developed to evaluate comparative long‐term safety under real‐world conditions. These methods include the experimental hierarchical approach, which extends the analytical follow‐up period for the test drug to include the time after switching away, and the overlapping approach, which extends the follow‐up period for both the test and comparator drugs to include the time after switching away. This paper uses multistate model methodology to consider initial and subsequent exposures in evaluating the estimators in these two methods. Our mathematical evaluations and simulations demonstrate that the estimators inflate the type‐1‐error across different switching and outcome incidence rate scenarios. Therefore, we propose a minor modification of the estimators to preserve the type‐1‐error. Currently used methods are simple but biased and lack clearly defined estimands. Methods based on multistate models may help identify and refine new estimands for evaluating long‐term safety.

## 1 Introduction

Long‐term safety evaluation is important to characterize the benefit–risk of many chronic indications with expected repeated use of a given treatment (drug) [1]. Marketing authorization holders or regulators often conduct observational studies designed primarily to answer long‐term safety and effectiveness questions to fill knowledge gaps that were not answered at the time of drug approval [2, 3]. For example, drug registries by CorEvitas Psoriasis Patient Registry [4] investigated the impact of patient characteristics and clinical factors on biologic drug switching of patients with psoriasis and psoriatic arthritis [5]. Unlike (short‐term) clinical trials that can tightly manage initiation and adherence to pre‐specified test and comparator drug for a fixed time period, long‐term observational studies are prone to have real‐world treatment initiation and adherence patterns. Those patterns include initiating a novel treatment, stopping treatment, gaps in treatment, and switching. Manitz et al. [6] describe different adherence patterns and emphasize the importance of defining an appropriate estimand dependent on the indication, possible adverse events and possible treatments. These patterns and the extent of utilization can be hard to predict, especially for newly approved classes of treatment. For illustration, we will consider evaluating long‐term risk in chronic indications treated with systemic biologics [7]. Patients with psoriasis are candidates for systemic therapy. Often, patients start with one drug and have to switch to another because they develop a drug tolerance or fail to maintain initial response [5, 8, 9]. Safety evaluations consist of comparing the adverse event (AE) free survival between the drug of interest and the comparative treatment. Handling different adherence patterns is one of the challenges for the analytical approaches to compare the AE‐free survival.

Several analytical approaches exist in comparative safety [10, 11, 12, 13]. This paper will focus on two commonly used approaches. More specifically, we review the experimental hierarchical and the overlapping approaches [10, 14, 15]. Both approaches use the Cox model to estimate a hazard ratio (HR) for measuring the treatment effect in a real‐world setting, accounting for treatment switch. The aim is to compare the risk for an AE between the drugs. However, if the AE happens after switching the drug, the cause for this AE may be either the first treatment or both. The two approaches handle this by attributing the follow‐up time and the possible AE to each initiated drug independent of the adherence. The follow‐up time and possible AE are attributed to the test drug (TD) or comparator drug (CD) in an asymmetric way in the experimental hierarchical approach and in a symmetric way in the overlapping approach. The experimental hierarchical approach assumes an after‐effect only of the TD, whereas the overlapping approach for both [10, 14, 15]. Without loss of generality, we use the term adverse event (AE) to indicate the clinical outcome of interest in a time to failure model. Possible AE are developing a malignancy, an infection, a major cardiovascular event, and others. In our case study, we focus on the time of occurrence of any first event.

This paper uses multistate model methodology to evaluate the mathematical properties of the estimators from each of the two approaches. For simplicity and to isolate the impact of switching from other sources of bias, we investigate the situation with one switching in the absence of confounding and censoring beyond administrative censoring. In this simple idealized scenario, we demonstrate that both methods inflate the type‐1‐error because the analytical methods “restart the clock”. This means that patients were cloned when they switched and the time is recounted from time 0, the initiation of a drug. We also propose new estimators that do not suffer from this source of bias. The theoretical results are confirmed with simulations under different scenarios of outcome incidence and treatment adherence.

## 2 Methods

This section, reviews the multistate model framework and explains the experimental hierarchical and the overlapping approaches using that methodology. Finally, we discuss the properties of these estimators and suggest an adaption.

### 2.1 Time to Adverse Event and Multistate Model Framework

**FIGURE 1:**
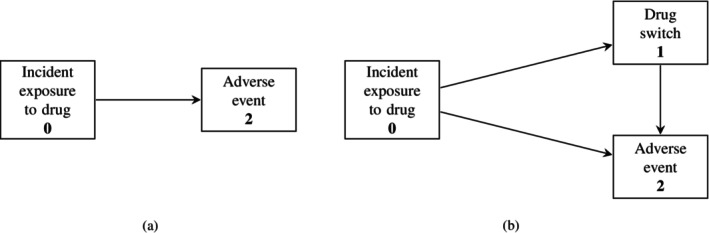
Schematic display, using multistate models, of the AE‐free survival setting and a three‐state model with exposure switch, called Illness‐death model. (a) The two‐state model with initial state incident exposure on treatment naïve patients and absorbing state of adverse event (AE). (b) The three‐state model: Illness‐death model without recovery. The initial state is the incident exposure, the interim state is a switch, and the absorbing state is the AE.

Assume a *time to failure scenario* where the initial state is the initiation or the first exposure to a drug treating the indication, and the failure is the first occurrence of the AE of interest. The outcome is considered as an absorbing state. In our motivating example, the initial state is entered at the time of initiation of a biological treatment, and the AE is malignancy. Let $X_{t}\in \left\{0,2\right\}$ be the state at time t, $X_{t-}$ the state occupied just before time t, and T the shortest time that $X_{t}\neq 0$, the time a patient is not in the initial state anymore: $T≔\inf\left\{t:X_{t}\neq 0\right\}$. Figure 1a gives the schematic display of the situation. The *hazard function* $\alpha \left(t\right)$ describes the instantaneous probability that an event happens in the next small time‐interval, given that no event has happened before t:

$$\begin{array}{c} \alpha \left(t\right)\cdot \mathrm{dt}=P\left(T\in \left(t,t+\mathrm{dt}\right)|T\geq t\right)\\ \;=P\left(X_{\left(t+\mathrm{dt}\right)-}=2|X_{t-}=0\right) \end{array}$$

The individual hazard $\alpha^{i}\left(t\right)$ is defined as the instantaneous probability that individual i has an event in the small time interval $\left[t,t+\mathrm{dt}\right)$ (dt an infinitesimally small time), given that individual i has not yet experienced an event before. In the standard time to failure scenario (see Figure 1a), a direct one‐to‐one mapping exists between the hazard and the probability to be event‐free: $P\left(T>t\right)=\exp\left(-\int_{0}^{t}\alpha \left(u\right)\mathrm{du}\right)$ [16].

The *cumulative hazard*, cumulative in time $\int_{0}^{t}\alpha \left(u\right)\mathrm{du}$, can be nonparametrically estimated in a sample of observations with the Nelson‐Aalen estimator. Assume $0<t_{1}<t_{2}<\ldots <t_{K-1}<t_{K}=t$ is the ordered sequence of observed failure times (or AE times) across subjects, the Nelson‐Aalen estimator is defined as

$$\hat{A}\left(t\right)=\sum_{k=1}^{K}\frac{\text{number of observed individuals failing}\;\mathrm{at}\;t_{k}}{\text{number of individuals without}\;\mathrm{AE}\;\text{just prior to}\;t_{k}\;\text{and under observation}}.$$

Comparing the (instantaneous) risk for an AE between two treatment groups (e.g., first exposure of the TD versus first exposure of the CD), the HR can be estimated with a *Cox model* and is parameterized by β.

The parameter β is assumed to be constant. If this assumption is invalid, [17] showed that the estimator $\hat{\beta }$ estimates a weighted average of the time‐dependent $\beta \left(t\right)$. The HR estimated from the Cox model $\exp\left(\hat{\beta }\right)$ approximately equals the ratio of the group specific Nelson‐Aalen estimators, that is, ${\hat{A}}_{\text{testdrug}}\left(t\right)/{\hat{A}}_{\text{comparator}}\left(t\right)\approx \exp\left(\hat{\beta }\right)$, if the proportional hazard assumption holds.

#### 2.1.1 Multistate Model: A Three‐State Model

In this section, we go beyond the two‐state model to introduce a new state, switching, or change in exposure. Figure 1b shows a specific multistate model, the Illness‐death model without recovery [18]; the three boxes represent three states, and the arrows represent a possible transition in time. This model consists of an initial state, an intermediate event, and an absorbing state. In our application, the initial state (state 0) is the first drug exposure or initiated drug, the intermediate event (state 1) is a switch from the initial drug exposure to another drug, and the absorbing state (state 2) is the AE. In this model, a patient can switch the drug before experiencing an AE. It is also possible for the patient to experience the AE directly after exposure to initial treatment only. $X_{t}\in \left\{0,1,2\right\}$ denotes the state an individual is in at time t and $X_{t-}$ is the state occupied just before time t. The cause‐specific hazards from state l to j, $l,j\in \left\{0,1,2\right\};\;l<j$ is defined via

(1) $$\alpha_{\mathrm{lj}}\left(t\right)\cdot \mathrm{dt}=P\left(X_{\left(t+\mathrm{dt}\right)-}=j|X_{t-}=l\right)$$

with dt an infinitesimal small time. The cumulative cause‐specific hazard can be estimated with the general Nelson‐Aalen estimator:

$${\hat{A}}_{l,j}\left(t\right)=\sum_{k=1}^{K}\frac{\text{number of observed}\;l\to j\;\text{transitions}\;\mathrm{at}\;t_{k}}{\text{number of individuals in state}\;l\;\text{just prior to}\;t_{k}\;\text{and under observation}}$$

In contrast to the standard time to failure model, the transition probability from state l to state j: $P_{\mathrm{lj}}\left(s,t\right)=P\left(X\left(t\right)=j|X\left(s\right)=l\right)$, s<t is a function of multiple cause‐specific hazards (1) and a direct interpretation of the results from the hazards on probability level is not possible [18].

In general, the Illness‐death model is assumed to be Markovian. That means that the transition out of a state does not depend on the entry time into this state or the sojourn time in this state. We refer to the discussion for non‐Markov models [19, 20].

### 2.2 The Experimental Hierarchical Approach

**FIGURE 2:**
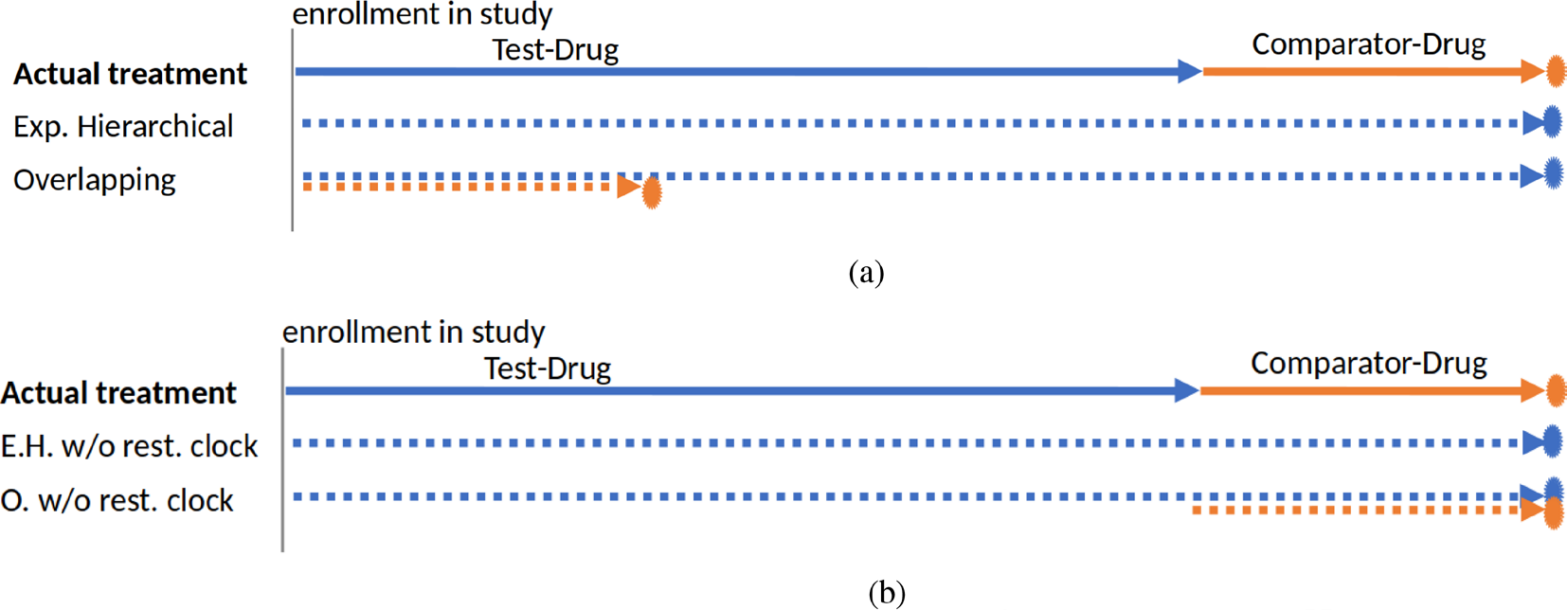
A possible patient journey (represents patient D in Appendix A). At study enrollment, the patient initiates and remains exposed to the test drug (TD) for a period (blue), then switches to the comparator drug (CD) and remain exposed for a period (orange). The follow‐up is censored before an AE occurs (circle). For this patient, the experimental hierarchical approach attributes the entire follow‐up time to TD while the overlapping approach clones the patient with one clone under TD and another under CD, giving the initiated exposure TD the entire follow‐up. (a) Time at risk and event with restarting the clock. (b) Time at risk and event without restarting the clock (modified approach).

**FIGURE 3:**
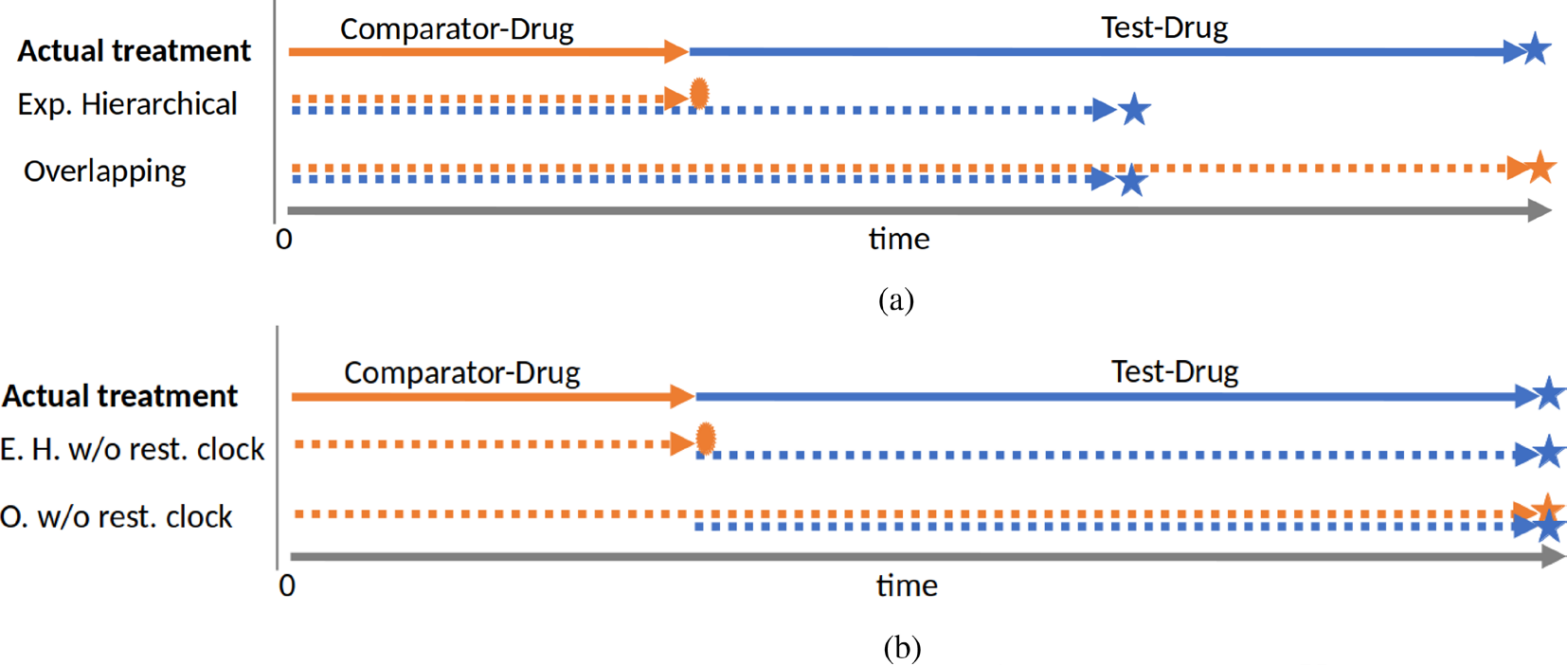
A possible patient journey (represents patient C in Appendix A). At study enrollment, the patient initiates and remains exposed to the comparator drug (CD) for a period (orange), then switches to the test drug (TD) and remain exposed for a period (blue). The follow‐up ends with observing an AE (star). The circle shows a censored event indicator. For this patient, the experimental hierarchical approach clones the patient, splitting the follow‐up time for each clone. The overlapping approach clones the patient with one clone under CD and another under TD, giving the initiated exposure CD the entire follow‐up. (a) Time at risk and event with restarting the clock. (b) Time at risk and event without restarting the clock (modified approach).

The experimental hierarchical approach aims to compare the AE‐free survival between two treatment groups, those exposed to TD and those exposed to CD [10]. The comparative safety estimator from this method is a HR fit by a simple Cox Model. However, to handle change in exposure due to switching, this model is not fit to the original data (one row per subject) with unique treatment assignment $Z\in \left\{\mathrm{TD},\;\mathrm{CD}\right\}$ and follow‐up for each patient but rather to augmented data with clones. More specifically, in the experimental hierarchical approach, follow‐up time and events after the initiation of the TD are attributed to the TD irrespective of switch away or whether the TD was the initial treatment. The follow‐up time after initiation of a CD is censored at switch away to the TD [10, 14, 15]. Figures 2a and 3a show two patient treatment journeys (patients adherence pattern) for patients switching drugs during their follow‐up. The arrows mark how the approaches assign the time at risk and the event indicator to the two drugs. Appendix A shows how the dataset needs to be structured to apply the Cox model and explains all possible patient journeys with examples. Figures 2a and 3a display patients D and C in the appendix.

A patient who switches from CD to TD contributes twice to the dataset for the same time period. The total follow‐up time of these two lines sums up to the total follow‐up for the original patient because the clock is restarted at the time of switch. This duplication of patients and time results in the same patient being at risk for an AE under both treatments at the same time. We will see with simulated data that restarting the clock causes a type‐1‐error if the hazards of the absorbing event are time‐dependent because the estimator combines hazard of incident users with the hazard of prevalent users. A simple improvement is not to restart the clock and anchor the start of follow‐up (time 0) to the initiation of the first drug. For the patients who switched, the second period (the clone) starts at the time of the switch, and the event time is the time of the AE since initiating the first drug. In other words, the patient's clone is treated like a left‐truncated observation [21, 22]. Figures 2b and 3b show possible patient journeys, including a treatment switch, and how the time at risk is analyzed without restarting the clock. These figures represent the same patients as in Figures 2a and 3a.

**FIGURE 4:**
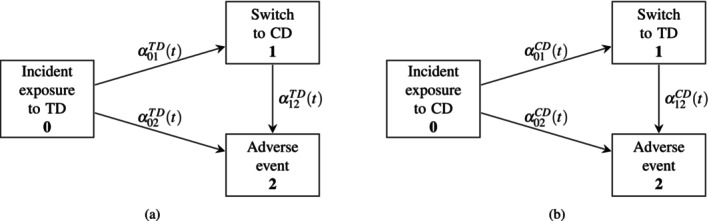
Schematic display of the three‐state Illness‐death models, applied to treatment switching and different initial drug. (a) Illness‐death model with initial state first exposure to test‐drug. (b) Illness‐death model with initial state first exposure to CD.

To understand the estimator of the experimental hierarchical approach better, we express the HR in terms of transition hazards. First, we go back to a time to failure setting, that is, only two states. Patients who start with the TD or the CD have the AE‐free survival hazard $\alpha^{\mathrm{TD}}\left(t\right)$ and $\alpha^{\mathrm{CD}}\left(t\right)$, respectively (Figure 1a with incident exposure TD and CD, respectively). Second, we include one possible state for treatment change/switching. The underlying model becomes an Illness‐death model. To facilitate further description below, we distinguish two models depending on the initial treatment (state 0) (Figure 4). Figure 4a,b shows transition hazard when the initiate state is the TD (respectively, the CD).

We assume a common switching time $t_{\mathrm{SW}*}$ for all switching patients for ease of notation. This might not be realistic in real life because patients may switch due to different individual reasons, for example, developing a drug intolerance or failing to maintain an initial response. We will use the Cox model to estimate the HR with the previously explained dataset for the experimental hierarchical approach. The HR expressed as a ratio of estimators for the hazards of the previously described two‐state and three‐state models is

(2) $$\hat{\mathrm{HR}}\left(t\right)=\frac{{\hat{P}}_{\mathrm{TD}}\left(t\right)\cdot {\hat{\alpha }}^{\mathrm{TD}}\left(t\right)+\left(1-{\hat{P}}_{\mathrm{TD}}\left(t\right)\right)\cdot {\hat{\alpha }}_{12}^{\mathrm{CD}}\left(t+t_{\mathrm{SW}*}\right)}{{\hat{\alpha }}_{02}^{\mathrm{CD}}\left(t\right)}$$

with ${\hat{P}}_{\mathrm{TD}}\left(t\right)$ the estimated probability of being a TD starter and event‐free at time (t) evaluated in the population of TD starters and prevalent users of TD at time t (initiated or switched to TD before time t and without an AE until time t). It is important to note that this probability is evaluated in the world where for the CD to TD switchers the clock is restarted at the time of entry into TD when talking about time t. $1-{\hat{P}}_{\mathrm{TD}}\left(t\right)$ is then the estimated probability of being a CD to TD switcher and event‐free at time (*t*) evaluated in the population just described. We refer to Appendix A for a step‐wise derivation from the heuristic to the mathematical formulation. The ratio is the event hazard from the TD initiators and those who switched to the TD divided by the event hazard of those who are the CD initiators and remain under CD during the whole follow‐up. Please note that the time is shifted back for the patients who switch to the TD. Counting time since study entry, this hence requires adding the time of switch for ${\hat{\alpha }}_{12}^{\mathrm{CD}}\left(t+t_{\mathrm{SW}*}\right)$.

In the real‐world, not all patients switch at the same time. The estimated transition hazard ${\hat{\alpha }}_{12}^{\mathrm{CD}}\left(t+t_{\mathrm{SW}*}\right)$, which is shifted by a common switching time, can be replaced by the (marginal) observable transition hazard arising from an empirical mixture,

(3) $$\frac{1}{\sum_{i=1}^{\#\mathrm{SW}}1\left(T_{i}\geq t\right)}\sum_{i=1}^{\#\mathrm{SW}}{\hat{\alpha }}_{12}^{\mathrm{CD}i}\left(t+t_{\mathrm{SW}}^{i}\right)$$

with $t_{\mathrm{SW}}^{i}$ the individual switching‐time for patient i, ${\hat{\alpha }}_{12}^{\mathrm{CD}i}\left(.\right)$ the estimated transition hazard of individual i, $i\in \left\{1,\ldots ,\#\mathrm{SW}\right\}$, with #SW the number of patients who will switch from CD to TD. This number #SW is known because we shift the time back and treat them as if the time of switch is the same initial time 0 as for the initiation of the first drug. Please note that a specific derivation of the failure hazards ($\alpha^{\mathrm{TD}}\left(t\right)$, $\alpha^{\mathrm{CD}}\left(t\right)$) in terms of the transition hazards of the Illness‐death model is a combination of all three transition hazards (see [23]).

We mentioned earlier that we possibly face an issue if one patient is at risk twice at the same time. First, it is unclear how the type‐1‐error is affected if one patient is counted twice in the first and not in the second timeframe (after the switch) [22]. Second, incident and prevalent users are mixed up, and the same patient is treated independently during the two observation periods. We suggested not restarting the clock. This would keep the chronology of events since a common initial state and time 0. In the equation above, this means that we do not add the time of switch in the estimated transition hazard ${\hat{\alpha }}_{12}^{\mathrm{CD}}\left(t\right)$, and the probability ${\hat{P}}_{\mathrm{TD}}\left(t\right)$ can be estimated in the real‐world (without restarting the clock). This ensures that patients contribute to the risk set when they are really at risk.

### 2.3 The Overlapping Approach

The overlapping approach also intends to compare the AE‐free survival under the two treatments with a HR. However, the patients' time at risk and events are assigned to the TD and CD differently compared to the experimental hierarchical approach. The overlapping approach attributes the follow‐up time and events of treatment‐naïve patients to the initiated drug irrespective of switch away. Moreover, the follow‐up time and events after a switch (the time and events of prevalent users) are also attributed to the drug the patient switched to [10, 14, 15]. Figures 2a and 3a show two patient treatment journeys and how the overlapping approach assigns the time at risk and the event indicator to the two drugs. Appendix A reviews the approach and illustrates patient treatment journeys and how data are structured to apply the Cox model for the overlapping approach.

By shifting the time at risk after a drug switch back to 0, a patient may appear at risk for an AE twice at the same time for both treatments. Please note that the overlapping approach, without restarting the clock, still uses the time period after the switch twice but counted since the first drug use (see also Figures 2b and 3b) [21, 22].

The HR resulting from the application of the overlapping approach with the multistate model framework can hence be expressed as follows:

(4) $$\hat{\mathrm{HR}}=\frac{{\hat{P}}_{\mathrm{TD}}\left(t\right)\cdot {\hat{\alpha }}^{\mathrm{TD}}\left(t\right)+\left(1-{\hat{P}}_{\mathrm{TD}}\left(t\right)\right)\cdot {\hat{\alpha }}_{12}^{\mathrm{CD}}\left(t+t_{{\mathrm{SW}}^{*}}\right)}{{\hat{P}}_{\mathrm{CD}}\left(t\right)\cdot {\hat{\alpha }}^{\mathrm{CD}}\left(t\right)+\left(1-{\hat{P}}_{\mathrm{CD}}\left(t\right)\right)\cdot {\hat{\alpha }}_{12}^{\mathrm{TD}}\left(t+t_{{\mathrm{SW}}^{*}}\right)}$$

with ${\hat{P}}_{\mathrm{TD}}\left(t\right)$ as above and with ${\hat{P}}_{\mathrm{CD}}\left(t\right)$ the estimated probability of being a CD starter and event‐free at time (t) evaluated in the population of CD starters and TD to CD switchers at risk at time t. This probability is evaluated in the world where the clock is restarted for the TD to CD switchers at the time of switch when talking about time point t. $1-{\hat{P}}_{\mathrm{CD}}\left(t\right)$ is then the estimated probability to be a TD to CD switcher and event‐free at time (t) evaluated in the population just described. The overlapping approach has a similarity in the numerator and denominator. Both parts combine the AE‐free survival hazard with the transition hazard shifted back to time 0. Suppose we do not assume a common switching time for all patients who will switch (independent of which drug). In that case, the transition hazard out of the intermediate state can again be expressed as a sum of individual hazards analogous to (3).

Not restarting the clock means that a common time 0 is maintained for all patients regardless of whether they change medication or not. Nevertheless, both estimators double the switched patients, and the increase in the number of patients affects the originally defined type‐1‐error level [24].

## 3 Simulations

We confirm the problem with restarting the clock using simulated data under multiple scenarios. These scenarios are all under the null hypothesis (i.e., no treatment difference) and include two extreme simulation scenarios with many observed events and five more realistic scenarios for rare events. The extreme scenarios help visualize the implications of the approach on the transition hazards estimators of the multistate model representation, which would be harder to see with fewer events. Scenarios with constant AE hazards are compared with time‐dependent Weibull distributed hazards to illustrate the implication of time dependence on the estimators. Safety evaluations on patients with psoriasis are characterized by only a few events. An AE can be, for example, a malignancy or an infection. The simulation specifications should mimic these real‐world data [25].

### 3.1 Simulation Specifications and Scenarios

#### 3.1.1 Extreme Scenarios

For each scenario described below, we simulate the event time T for 3000 patients starting with the TD and 3000 patients starting with the CD. Administrative censoring is always taken as 8 years. We run 1000 simulations. The AE‐hazard is taken as $\alpha \left(t\right)=4$, that is, constant, in scenario 1 and a Weibull‐hazard with shape = 0.3 and scale = 2 in scenario 2. These scenarios result in about 5200 and 4700 observed events, respectively. We choose a constant switching time of 1 year and a switching proportion of 30%, randomly generated independently of the time to failure. Therefore, patients with an event before 1 year could be “theoretical switchers” but not “observed switchers”, and the observed proportion of switchers was, on average, less than 30%. The observed switching proportion is a random experiment if the patient is still at risk for an event and can switch or not.

#### 3.1.2 Type‐1‐Error and Bias Evaluation

The same settings are taken as above. In addition, for the type‐1‐error evaluation under the null hypothesis, we select shape and scale Weibull parameters of the time to failure such that the total number of events is the same at the end of the follow‐up period across scenarios: about 330 events. This corresponds to an actual situation of relatively rare events. More specifically, the parameters are the following:

- a constant hazard with shape 1 and scale 142.85
- Weibull distributed hazard with shape 1.1 and scale 109.93
- Weibull distributed hazard with shape 1.2 and scale 88.36
- Weibull distributed hazard with shape 1.3 and scale 73.45
- Weibull distributed hazard with shape 1.5 and scale 54.66

We consider two switching times, 1 and 5 years, and different switching proportions, 25% and 50%. Table 1 gives an overview of the different simulation scenarios.

**TABLE 1:** Simulation scenarios for 3000+3000 simulated patients and an administrative censoring after 8 years.

Scenario	Hazard shape	Hazard scale	SW.‐ time	Switching proportion
1	1	4	1	30%
2	0.3	2	1	30%
3	1	142.85	1	25%
4	1	142.85	5	25%
5	1	142.85	1	50%
6	1	142.85	5	50%
7	1.1	109.93	1	25%
8	1.1	109.93	5	25%
9	1.1	109.93	1	50%
10	1.1	109.93	5	50%
11	1.2	88.36	1	25%
12	1.2	88.36	5	25%
13	1.2	88.36	1	50%
14	1.2	88.36	5	50%
15	1.3	73.45	1	25%
16	1.3	73.45	5	25%
17	1.3	73.45	1	50%
18	1.3	73.45	5	50%
19	1.5	54.66	1	25%
20	1.5	54.66	5	25%
21	1.5	54.66	1	50%
22	1.5	54.66	5	50%

The Weibull‐parametrization is done according to the R‐function rweibull() from the package stats, version 4.3.2. Type‐1‐error evaluation is done under the null hypothesis (equal AE‐hazards) by fitting the Cox model with the datasets adapted according to the two approaches and testing the null hypothesis (β=0) with the Wald test [26]. Running 1000 simulations, we would expect at a type‐1‐error rate of 5%, approximately 5% of the simulation scenarios, to have a *p*‐value below 0.05, such that the null hypothesis of β=0 can be rejected. If the percentage exceeds 5%, we call this a type‐1‐error inflation.

### 3.2 Results of the Simulations

#### 3.2.1 Extreme Scenarios

**FIGURE 5:**
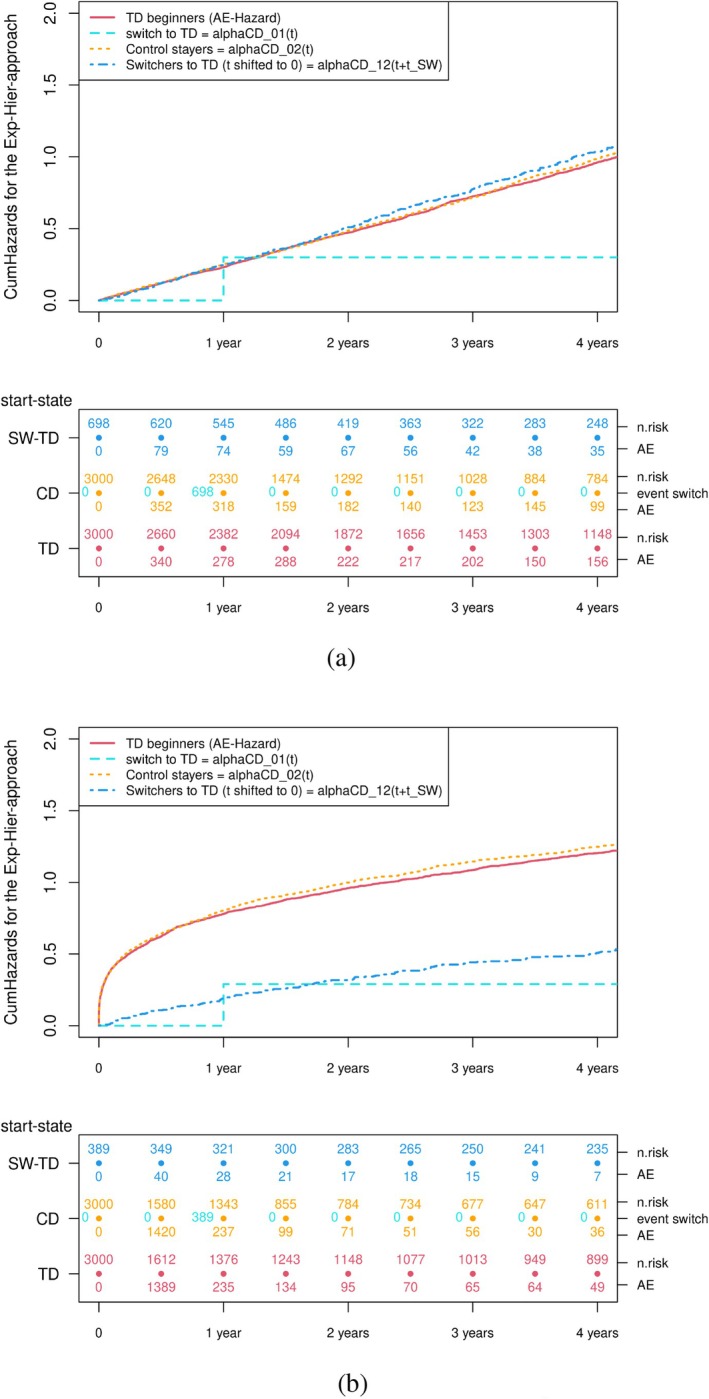
Nelson‐Aalen plots of the cumulative transition hazards from the multistate model representation of the experimental hierarchical approach. (a) Scenario 1: constant failure‐hazard α = 4 (shape = 1 and scale = 4). (b) Scenario 2: Weibull distributed failure‐hazard with shape = 0.3 and scale = 2.

In the first two scenarios, Figure 5 shows the Nelson‐Aalen estimates for the cumulative hazards for the experimental hierarchical approach. Per Equation (2), the numerator consists of the failure‐hazard for patients starting TD at study entry (cumulative Nelson‐Aalen estimator in red) combined with the patients switching from CD to TD, that is, the transition hazard of those who switched to the TD (cumulative Nelson‐Aalen estimator in dark blue). The cumulative hazard involved in the denominator (CD non‐switcher and CD initiator until switch) is shown in orange. The cumulative hazard in light blue quantifies the cumulative switching hazard from the CD to the TD. Figure 5a shows simulation results from scenario 1 with a constant simulated failure hazard. In that scenario, all estimates of the cumulative transition hazards have the same slope. However, at time 0, the number at risk includes not only the 6000 simulated patients but also 682 clones.

**FIGURE 6:**
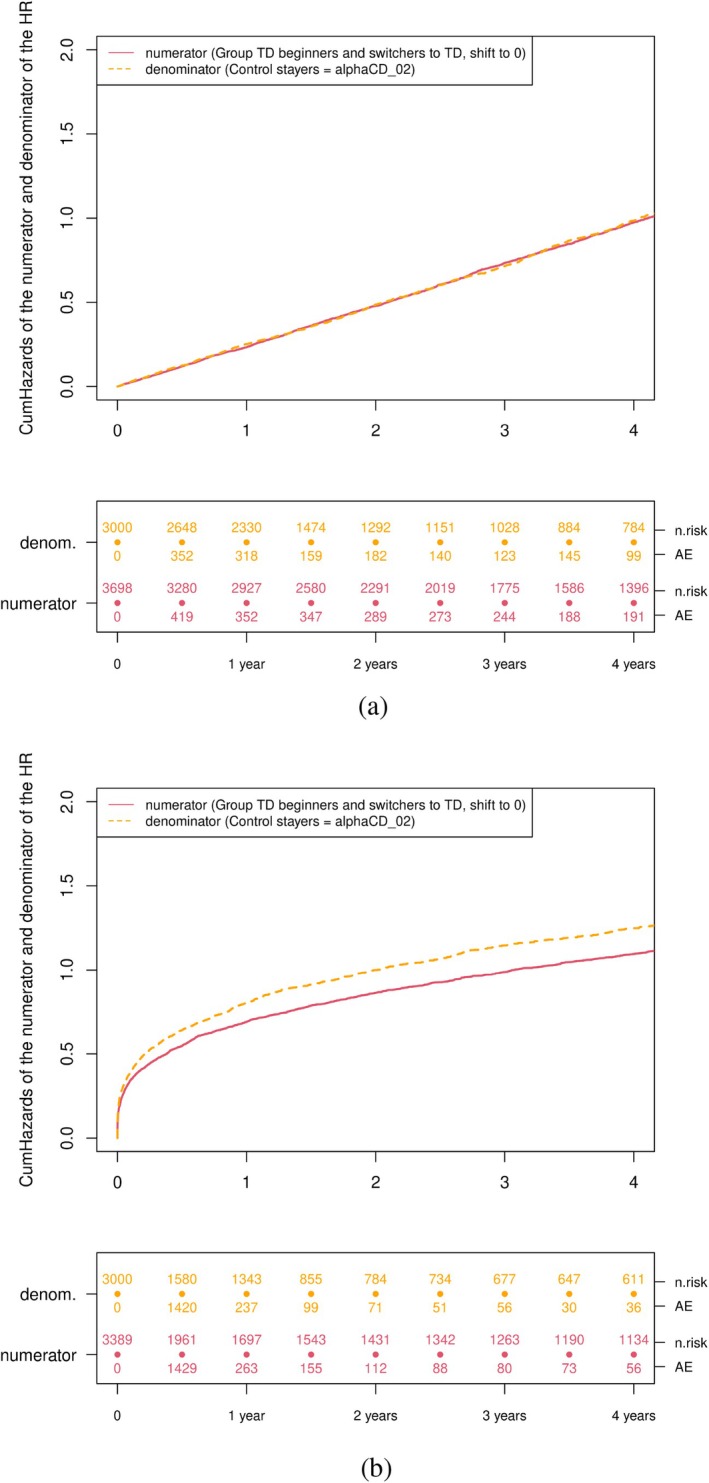
Nelson‐Aalen plots of the transition hazards from numerator and denominator (denom.) of the HR representation (2) of the experimental hierarchical approach. (a) Scenario 1: constant failure‐hazard α = 4 (shape = 1 and scale = 4). (b) Scenario 2: Weibull distributed failure‐hazard with shape = 0.3 and scale = 2.

Figure 5b shows simulation results from scenario 2 with a Weibull failure‐hazard, therefore time‐varying rather than constant. In this scenario, it appears very clearly that the switcher contribution, for whom the slope of the cumulative failure hazard differs from the slope of the cumulative failure for patients starting with TD, will greatly affect the overall estimate when combining them for the numerator of the estimator. This will be the case in each scenario for which follow‐up is shifted back to time 0 for those who switch from CD to TD, and the failure hazard is time‐dependent. Figure 6 shows the Nelson Aalen estimates of the cumulative numerator and the denominator of the estimator (2) to visualize this. The denominator is the previous orange curve as in Figure 5b, and also orange in Figure 6. The numerator is the combination of the previous blue and red curve (displayed in red in Figure 6).

**FIGURE 7:**
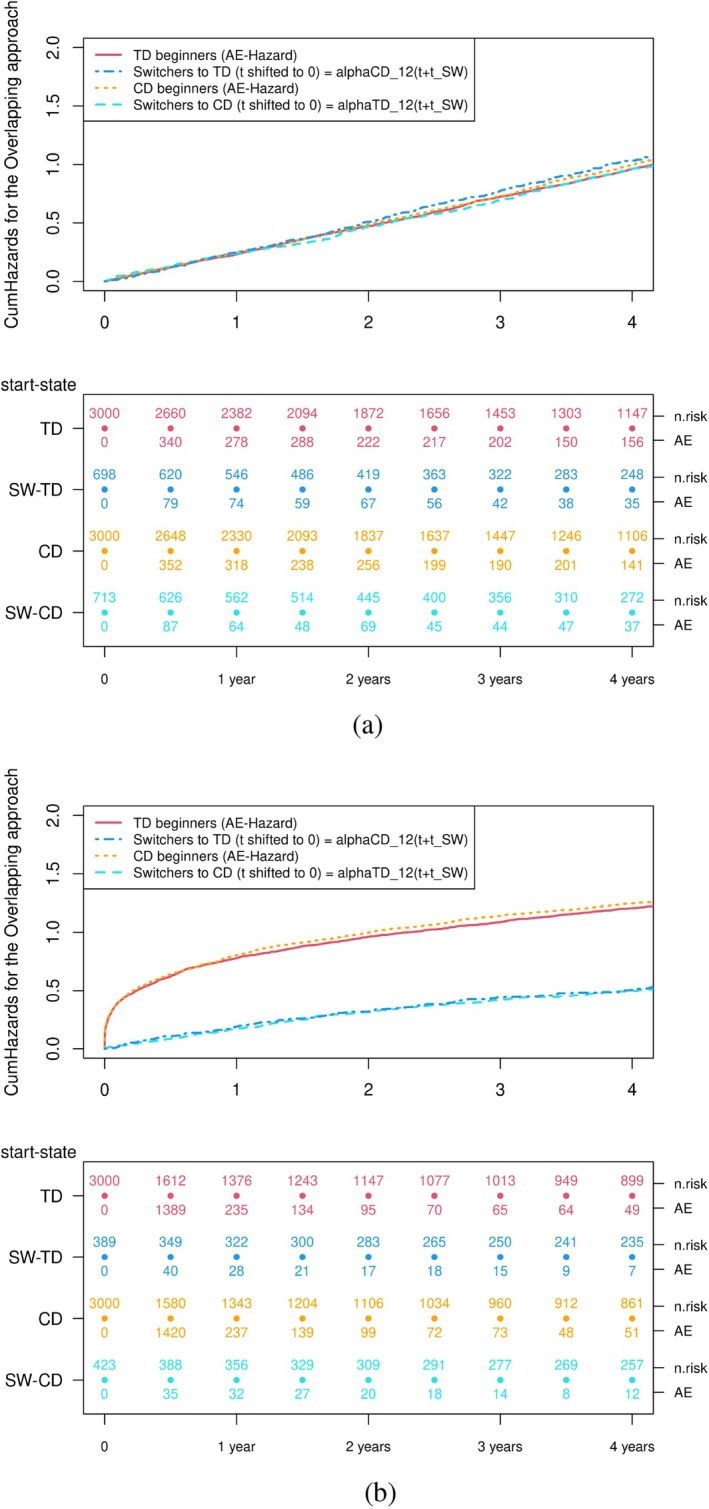
Nelson‐Aalen plots of the transition hazards from the multistate model representation of the overlapping approach. (a) Scenario 1: constant failure‐hazard α = 4 (shape = 1 and scale = 4). (b) Scenario 2: Weibull distributed failure‐hazard with shape = 0.3 and scale = 2.

The Nelson‐Aalen estimates of the cumulative hazard resulting from applying of the overlapping approach are shown in Figure 7. As shown in Equation (4), the AE‐hazard for the TD starters and the hazard of those who switched to the TD contribute to the numerator (Nelson‐Aalen estimates in red and dark blue). The denominator consists of the AE‐hazard of the CD starters and the hazard from those who switched to the CD (Nelson‐Aalen estimates in orange and light blue). Since the hazards are constant, the transition hazards are the same independent of the time origin (initial 0 vs. reset to 0 for switchers). For a time‐dependent AE‐hazard, we can observe that the slope of the two estimated cumulative transition hazards which will be combined in the overlapping approach is not the same. Because the overlapping approach has a symmetry as shown in Equation (4), and we have simulated the same number of TD and CD starters and switchers in both groups, even though the numerator and denominator combine each two hazards with a different origin, their combination is only in this special case equal and in theory not. The at‐risk table shows that the approach doubles the number of patients switching treatment.

#### 3.2.2 Type‐1‐Error and Bias Assessment

**FIGURE 8:**
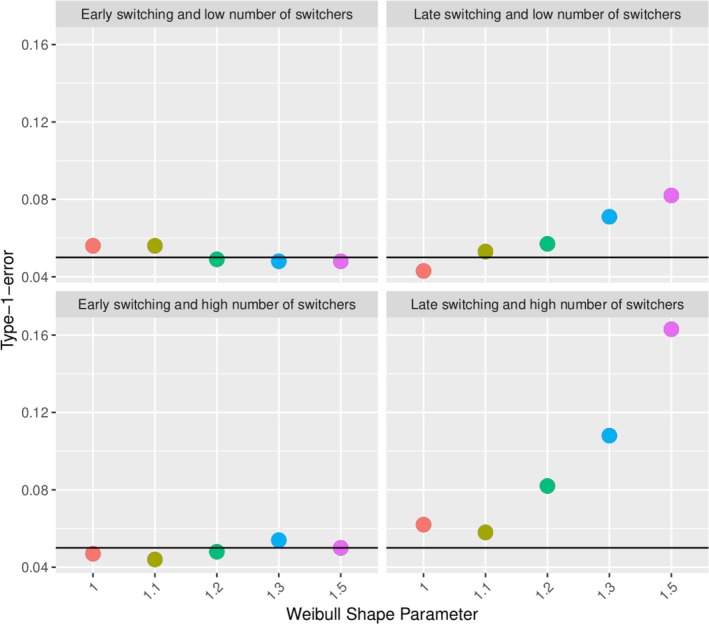
Type‐1‐error inflation of the experimental hierarchical approach in different scenarios (varying shape parameter (horizontal axis in each panel), switching proportion (top panels low: 25%, bottom panels high: 50%), and time of switching (left panels for early switch at 1 year, right panels for late switch at 5 years)).

Scenarios 3‐22 investigate the impact of the proportion of switchers as well as the time of switch in simulations when the events are relatively rare. Figure 8 shows the type‐1‐error inflation (the percentage of rejections of the null hypothesis of equal treatment from 1000 simulations) for the experimental hierarchical approach with two switching proportions (25% top and 50% bottom) and a fixed early (after 1 year, left) and late (after 5 years, right) switching time. The late switching time causes a higher type‐1‐error inflation for the scenarios with a time‐dependent AE‐hazard. This is increased if the hazard is strongly time‐dependent and the switching proportion is higher.

**FIGURE 9:**
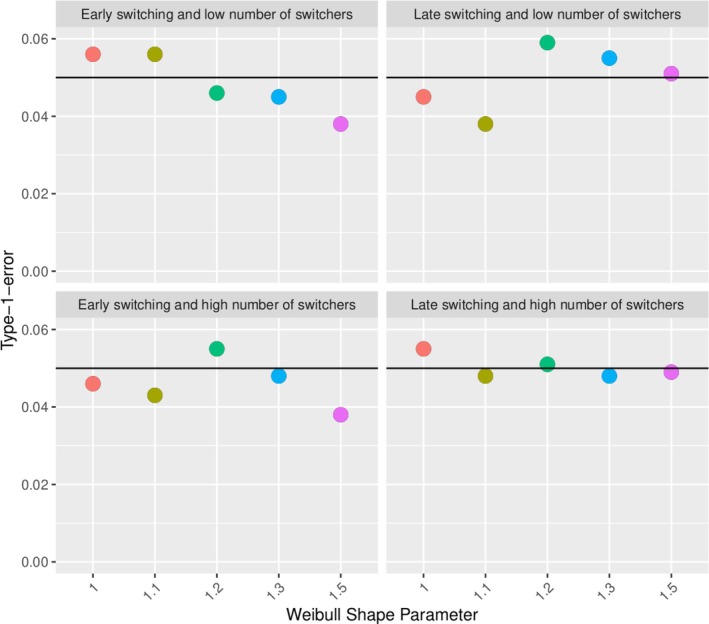
Type‐1‐error evaluation of the modified experimental hierarchical approach w/o restarting the clock for the switchers in different scenarios as in Figure 8.

Considering a variation of the experimental hierarchical approach, we apply the above principles without restarting the clock, that is, we maintain the time origin fix. Figure 9 shows the type‐1‐error inflation of the same simulation scenarios as in Figure 8. Restarting the clock is the source of the type‐1‐error inflation.

**FIGURE 10:**
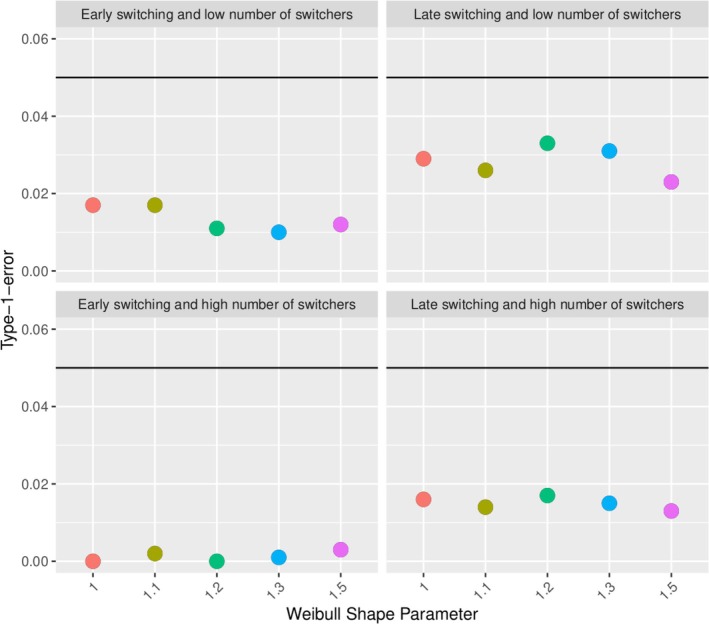
Type‐1‐error evaluation of the overlapping approach in different scenarios as in Figure 8.

Estimating the overlapping approach with the same simulation settings as the experimental hierarchical approach, we see that the percentage of rejections is below the nominal level of 0.05 (see Figure 10). The doubling of patients causes fewer rejections of the null hypothesis, a deflation of the type‐1‐error. This is also confirmed by a variation of the overlapping approach without restarting the clock for the switchers (Figure in Appendix C).

## 4 Discussion

We used multistate models to investigate the properties of two commonly used estimators in long‐term comparative safety. Our mathematical derivation and our simulations uncovered that these estimators re‐start the clock, resulting in a type‐1‐error inflation under the null when the hazard is not constant in time. Under the null, the bias is away from the null with type‐1‐error inflation in multiple safety‐relevant scenarios. We also propose an improvement of these estimators, not restarting the clock, that results in a slight improvement in preserving type‐1‐error. We used the plots of the Nelson‐Aalen estimator to visualize the impact of restarting the clock and cloning.

The main problem of shifting time is that the observations are shifted, but the hazard function in the Cox model is not shifted. If the original hazard is constant in time, in the same way in both treatment groups, then shifting time while fitting the Cox model with the same original risk does not inflate the type‐1‐error. However, if the original hazard is not constant or is different in the two treatments, shifting time without readjusting the original hazard can be problematic. Therefore, applying left‐truncation methods, stratifying the Cox model by time of entry into the cohort relative to drug initiation, or adjusting for time of entry [1, 11, 27] can improve this restarting the clock issue. Maintaining the chronology of events relating to exposure and outcome is recognized by many epidemiologists, for example, Suissa et al. [11] and Gagne [28].

The experimental hierarchical approach leads to bias beyond the scenarios under the null. It estimates the comparative risk in an asymmetric conservative way, always attributing more risk to TD. Thus, using the formulas we derived in this paper, we can also see that the bias is toward the null under the alternative of a protective effect of TD. A possible protective effect of the TD is blurred by including the TD switchers in the at risk set.

In addition, the overlapping approach also leads to bias beyond the scenarios under the null. This approach is symmetric in the cloning, and the two treatment groups are cloned similarly after switching. Thus, our simulation shows less type‐1‐error inflation than the hierarchical approach when the amount and timing of the switch are the same in the two treatments. However, our mathematical derivation indicates a bias in the overlapping approach in other scenarios because this method also combines prevalent and incident users. For example, consider the hypothetical example of two patients, one starting with the TD and the other with the CD. Both patients switch at the same time and have the same AE‐hazard and cause specific transition hazards in the Illness‐death model. Assume that the transition hazard from the intermediate (switched drug) state to the AE at time $t+t_{\mathrm{SW}}$ is higher than the transition from the initial (initial drug) state to the AE at time t. This could be because a switch of the drug is harmful to the patient. This harmful effect of a treatment switch cannot be detected when switching is symmetric and occurs at the same time in both treatment groups.

Additionally, failing to account for the correlation between a subject and his clone in the data at the same time can lead to bias. In both approaches, all or some of the patients who switch are cloned and are in the dataset twice at the same time t, whereas the fitted Cox model in these approaches does not account for this correlation. Therneau et al. [22] describe this with “This however is almost always a data error, since it corresponds to two copies of the subject being present in the same strata at the same time, e.g., she could meet herself at a party”. That will impact study power and type‐1‐error [29, 30, 31]. Therefore, when using either of these methods or even our proposed correction for the overlapping approach, we recommend correcting for correlation and number of cloned patients in the estimation of risk. For example, sandwich estimation or bootstrapping of the original patients can help to estimate a standard error that better reflects true risk uncertainty.

In our simulation scenarios under the null, the switching pattern was simple. The estimator is identified under both approaches, which simplifies the evaluation of the type‐1‐error. A potential extension of our work would be to fully use the Illness‐death model process to simulate the time to failure outcome as well as the switch with the algorithm in [18]. For example, the R‐package simIDM [32] would allow more complex switching patterns under the null‐ as well as investigate estimator properties under some alternative hypotheses. We give a brief summary in Appendix B and see that these extensions confirm what we already observed in our simple scenarios. Another extension would be to consider multiple switching events or a competing risks setting.

Beyond the null, identifying the target causal estimand in these approaches is difficult. The overlapping and hierarchical approaches attempt to use the Cox model to fit an “ever” versus “never” comparison of a long‐term safety outcome accounting for switching. Nevertheless, the status of “ever” and “never” user of a treatment is time‐varying as a patient could be a “never” user at one time and become an “ever” user. In these situations, we could not map the estimator resulting from these methods to an estimand from a hypothetical comparative randomized trial anchored at a given time across patients.

Other approaches as reviewed in [12, 33], have clearer causal estimands, such as the structural nested failure time and the marginal structural models that handle change in exposure with time‐varying confounding. However, whether those estimands are well‐suited to answer questions about long‐term safety (e.g., 8 to 10 years) when switching is common and happens within 1 to 2 years is debatable. For example, implementing the marginal structural model to account for switching and long‐term risk would typically censor patients who switch and up‐weight patients remaining on the initiated drug that are more similar than those who switched using inverse probability of censoring weights. This implementation would target the hypothetical estimand of the comparative risk had everyone in the study remained on the initiated drug. The estimators may be unstable with increasingly large weights over time on very few patients. Also, more importantly, the meaning of this hypothetical estimand is challenging to justify clinically when nearly no one remains on an initiated drug for as long as 10 years, that is, the positivity assumption is violated. Recently, Buehler et al. [34] also used multi‐state models to understand estimands in clinical trials. The authors advocate using interpretable target marginal causal estimands to account for real‐world patterns such as switching.

Quantifying long‐term risk and refining our questions of interest in post‐market safety is important for patients and public health. The currently used methods are simple, biased, and do not have clearly defined estimands. As authors in [34] concluded with clinical trials, we also consider with observational studies that the methods in multistate models and addressing complex causal inference questions may help to identify and refine new estimands and direct to new methods and tools to estimate them. The setting of several treatment switches can be visualized with an Illness‐death model with recovery or a progressive multistate model. Nonparametric and semiparametric estimators exist in both models, even if the Markov assumption is not fulfilled [19, 20, 35]. A violation of the Markov assumption would be if the risk of an AE can also depend on how long the patient already got the treatment or the number of previous treatment switches. The multistate model thinking helps us to identify potential pitfalls of the two approaches and can help to define new estimands to measure a treatment effect when treatment switches are common.

## Ethics Statement

The authors have nothing to report.

## Conflicts of Interest

The authors declare no conflicts of interest.

## Supporting information


**Appendix S1:** Supporting Information.

## Data Availability

R‐files generating the simulation datasets are available in the online Supporting Information.
