# Supplementary material for: Long‐Term Safety Evaluations in the Presence of Switching: Evaluation of Two Approaches
Source: Pharm Stat. 2025 Sep 26;24(6):e70039. doi: 10.1002/pst.70039 (PMC12475889; doi:10.1002/pst.70039)
Supplement: Supplementary file 1 — Appendix S1: Supporting Information. [file PST-24-0-s001.pdf]

Long-term safety evaluations in the presence of switching: evaluation of two approaches

Sandra Schmeller[1], Rima Izem[2], Pedro Lopez Romero[3] and Valentine Jehl[2]
July 16, 2025

[1] Institute of Statistics, Ulm University, Germany
[2] Novartis Pharma AG, Basel, Switzerland
[3] Novartis Pharma AG, Madrid, Spain

1 Appendix A

The experimental hierarchical approach

Patients initiating TD at study entry were assigned to the TD the whole follow-up time, regardless of a possible switching to the CD. Patients initiating CD at study entry were assigned to the CD until switching, the occurrence of an event or censoring, whatever occurs first. If the patient switches to the TD, the follow-up time for the CD is censored at the time of switch. Then a clone is generated at switch time with follow-up time assigned to TD. However, for the clone's follow-up, the clock is restarted with the entry time set to 0 (instead of the time of switch) leading to the clone's follow-up period after the switch to be shifted back in time relative to the original patient's follow-up. This clone is again considered at risk for an event until event occurrence or censoring. Using four patient treatment journeys, table 1 illustrates how the dataset needs to be structured to apply the Cox model using the experimental hierarchical approach. Patient A enters the study at the time of TD initiation and has an AE after 5 years. We assume that patient A does not switch the drug and therefore the event and the whole time at risk ($T = 5$) are assigned to the TD. Patient B enters the study at the time of CD initiation and experiences an event after 6 years without switching to the TD. The event and time at risk are assigned to CD. Patient C enters the study at the time of CD initiation, switches after two years of treatment from the CD to the TD, and suffers an event 1 year after the switching. This patient contributes with two periods translating in two lines in the dataset. The first represents the time under the CD, for which no event is counted, and the second, the time under the TD. Note that the time under TD is shifted back to time 0 for the patient's clone. Patient D starts with the TD and switches after seven years to the CD and is censored after an additional year. The whole follow up of 8 years is considered to be under the TD, because a switching out of the TD is ignored by the experimental hierarchical approach. If the patient would suffer an AE after 8 years the patient trajectory would be the same as in table 1 just changing the event indicator from 0 (censored) to 1 (AE).

Patient	Start time	Stop time	Event indicator	Treatment
A	0	$T = 5$	2	TD
B	0	$T = 6$	2	CD
C period 1	0	$SW = 2$	0	CD
C period 2	0	$T - SW = 1$	2	TD
D	0	$T = 8$	0	TD

Table 1: Example dataset for the Cox-model for the experimental hierarchical approach. Event indicator =2 indicates the patient experienced the event, Event indicator =0 indicates a censored observation, SW is the switching time, T the time of the AE, TD: Test-Drug, CD: Comparator-Drug

From heuristic to the mathematical formulation:
To explain the experimental hierarchical approach we go step-wise from the heuristic to the mathematical formulation:

$$
\begin{aligned}
HR(t) &= \frac{\text{AE-hazard from TD initiators and those who switched to the TD}}{\text{AE-hazard being under CD of the CD initiators}} \\
&= \frac{\text{Mixture of the AE-hazard from TD initiators } (t) \text{ and the AE-hazard who switched to TD } (t + t_{SW}*)}{\text{AE-hazard under CD of the CD initiators } (t)} \\
&= \frac{\text{Mixture of } \alpha^{TD}(t) \text{ and } \alpha_{12}^{CD}(t + t_{SW}*)}{\alpha_{02}^{CD}(t)}
\end{aligned}
$$

The first fraction describes heuristically the numerator and denominator of the HR in words as in [1] (see main document). Realizing that the referred time differs between both Illness-death models in Figure 5 a) and b), further notations are required as introduced in the second step. The time t is counted since the initial state, the first exposure to a drug of treatment naïve patients. Combining the TD initiators with those who switched, the time of the second group is shifted back. In terms of the hazard notation, we have to add the time of switch. We refer to this as ”restarting the clock”. In the third line, we expressed the hazards with the previously introduced notation for the hazards.

The overlapping approach
The whole follow-up and possible event are assigned to the initial drug regardless of any switches. Furthermore, the exposure time and event after a switch is additionally attributed to the drug switched into. Possible multiple contributions per patient due to the doubling of patients are treated independently. For the second contribution, the time is shifted back to 0. Table 2 illustrates patient treatment journeys and how data are structured for the overlapping approach.

Patient	Start time	Stop time	Event indicator	Treatment
A	0	$T=5$	2	TD
B	0	$T=6$	2	CD
C period 1	0	$T=3$	2	CD
C period 2	0	$T-SW=1$	2	TD
D period 1	0	$T=8$	0	TD
D period 2	0	$T-SW=1$	0	CD

Table 2: Example dataset for the overlapping approach. Event indicator =2 is an observed AE, event indicator =0 is censored, SW is the switchingtime, T the time of an AE, TD: Test drug, CD: Comparator drug

Patients A and B start with the TD and CD and suffer an event after five and six years respectively without switching. Patient C starts with the CD, switches after two years, and experiences an event after a total of three years. This patient hence contributes twice to the dataset: period 1 attributed to the CD, with time at risk of three years and an event; period 2 attributed to the TD, with time at risk of one year and an event. Patient D starts with the TD and switches after seven years to the CD and is censored after an overall follow-up of eight years. This patient also is present in the data with two contributions: period 1 attributed to the TD, with time at risk as eight years; period 2 attributed to the CD, with time at risk as one year. Would this patient suffer an AE after the 8 years the event is counted twice, once for the first line and once for the second

line. The patients who switch contribute with their total follow-up time to the initial drug and in addition with the time from switch to the event/censoring to the subsequent drug. For the second contribution, the time period is shifted back to time 0.

From heuristic to the mathematical formulation:

We go step-wise from the heuristic to the mathematical formulation for the overlapping approach (analogous as before):

$$\begin{aligned} HR &= \frac{\text{AE-hazard from TD initiators and those who switched to the TD}}{\text{AE-hazard from CD-initiators and those who switched to the CD}} \\ &= \frac{\text{Mixture of the AE-hazard from TD initiators } (t) \text{ and the AE-hazard who switched to TD } (t+t_{SW}*)}{\text{Mixture of the AE-hazard from CD initiators } (t) \text{ and the AE-hazard who switched to CD } (t+t_{SW}*)} \\ &= \frac{\text{Mixture of } \alpha^{TD}(t) \text{ and } \alpha_{12}^{CD}(t+t_{SW}*)}{\text{Mixture of } \alpha^{CD}(t) \text{ and } \alpha_{12}^{TD}(t+t_{SW}*)} \end{aligned}$$

The overlapping approach has a similarity in the numerator and denominator. Both parts combine the AE-hazard with the hazard of those who switched the treatment, shifted back to time 0.

2 Appendix B

To be able to have more flexible switching times we simulate an Illness-death model according to [2] (see main document), p. 173. First, a competing risk model is simulated. The event times of a state 0 to state 1 transition and state 0 to state 2 transition is generated with probability $1-\exp(-\int_0^t \alpha_{01}(u)+\alpha_{02}(u)du)$. The type of event (i.e. switching or AE) is generated with a binomial experiment with probability $\alpha_{01}(t)/\alpha_{01}(t)+\alpha_{02}(t)$. Afterwards, the 1 to 2 transition is modeled and the event times are distributed according to $1-\exp(-\int_0^t \alpha_{12}(u)du)$. The data for the Cox model is again created according to the tables 1 and 2. We simulate two scenarios under the null, i.e. $\alpha_{12}(t)=\alpha_{02}(t)$ ([3]), one with constant and one with Weibull distributed transition hazards. We investigate various switching proportions by using different switching hazards $\alpha_{01}(t)$. The transition hazards are shown in table 3. Each scenario is simulated with an exponentially distributed censoring with parameter in $\{0.1,0.2,0.3,0.4,0.5\}$, and administrative censoring is done after 8 years with an accrual time of 4 years.

H0	$\alpha_{01}(t)$	$\alpha_{02}(t)$	$\alpha_{12}(t)$
Scenario 1 - App.B	$0.03, 0.05, 0.07, 0.09, 0.11$	0.08	0.08
Scenario 2 - App.B	scale= $0.05, 0.1, 0.15, 0.2, 0.25$, shape= 0.4	scale= 0.1, shape= 2	scale= 0.1, shape= 2

Table 3: Specified transition hazards for the simulation.

Figure 1 (a) and (b) shows that with different switching and censoring proportions the type-1-error of the experimental hierarchical approach is inflated when the hazards to state 2, the AE, are time dependent. This confirms the previous results. Also, not restarting the clock helps to keep the 5% level (not shown). The results for the overlapping approach are analogous to the results in section 4 and not shown here.

THIS LINE IS FOR THE REVIEWER-PDF-CREATOR. (IT CUTS THE FIGURES OTHERWISE.)

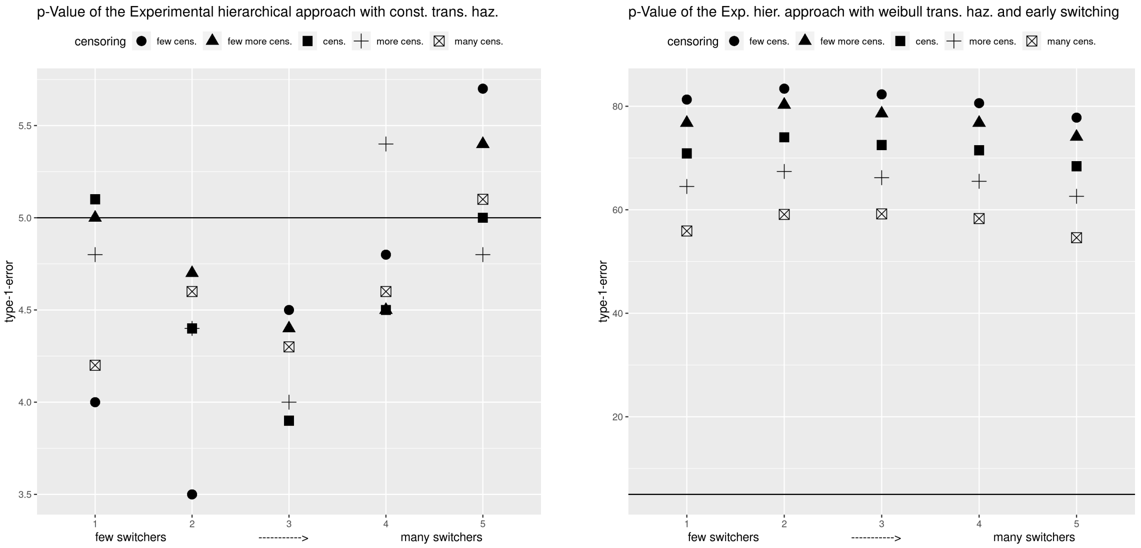

(a) Scenario 1 - AppB: Type-1-error evaluation with constant transition hazards and different switching and censoring proportions.

(b) Scenario 2 - AppB: Type-1-error evaluation with time-dependent transition hazards and different switching and censoring proportions.

Figure 1: Type-1-error evaluation of the experimental hierarchical approach.

To investigate the two approaches under the alternative, this simulation strategy is more suitable because the transition hazards $\alpha_{12}(t)$ and $\alpha_{02}(t)$ can be specified independently in the two groups starting with TD, or CD. Nevertheless, we refer to the discussion to debate the drawbacks of the two approaches.

3 Appendix C

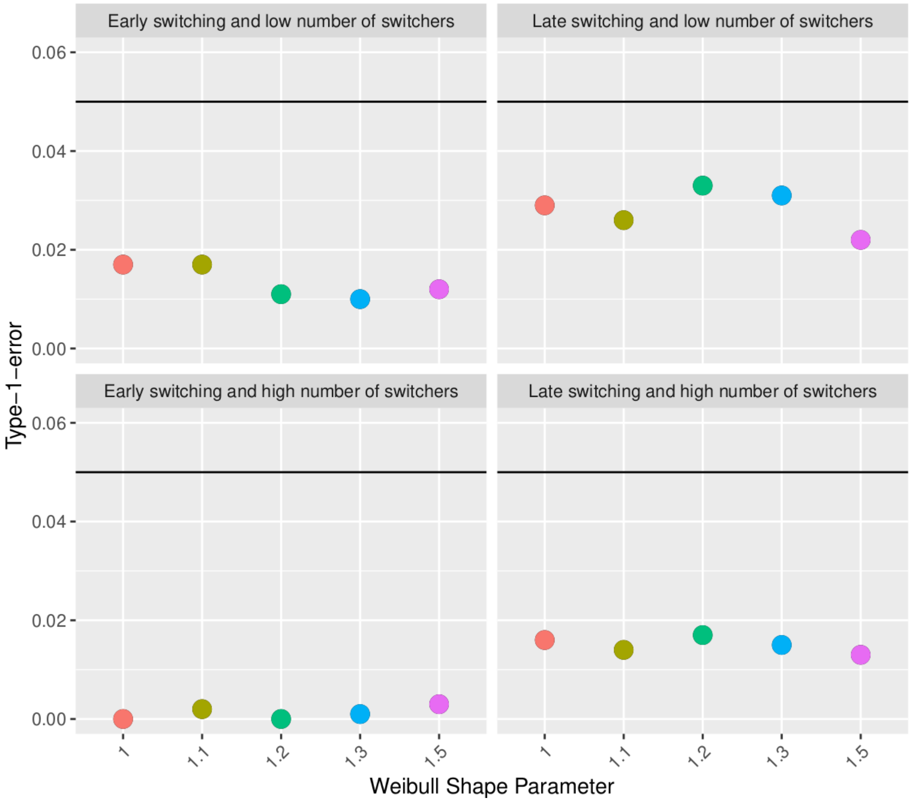

Figure 2: Type-1-error evaluation of the overlapping approach w/o restarting the clock for the switchers.

References

[1] Kremer Joel M, Bingham III Clifton O, Cappelli Laura C, et al. Postapproval Comparative Safety Study of Tofacitinib and Biological Disease-Modifying Antirheumatic Drugs: 5-Year Results from a United States–Based Rheumatoid Arthritis Registry *ACR Open Rheumatology.* 2021;3:173–184.

[2] Beyersmann Jan, Allignol Arthur, Schumacher Martin. *Competing risks and multistate models with R*. Springer Science & Business Media 2011.

[3] Meller Matthias, Beyersmann Jan, Rufibach Kaspar. Joint modeling of progression-free and overall survival and computation of correlation measures *Statistics in Medicine.* 2019;38:4270–4289.
